# IL-1 family cytokines in inflammation and immunity

**DOI:** 10.1038/s41423-025-01358-8

**Published:** 2025-10-14

**Authors:** Cecilia Garlanda, Irene Di Ceglie, Sebastien Jaillon

**Affiliations:** 1 IRCCS- Humanitas Research Hospital, Rozzano, Milan, 20089 Italy; 2 Department of Biomedical Sciences, Humanitas University, Pieve Emanuele, Milan, 20072 Italy

**Keywords:** inflammation, innate immunity, myeloid cells, Lymphoid cells, Immunopathology, Cytokines, Inflammatory diseases

## Abstract

Interleukin-1 (IL-1) was the first interleukin identified as a potent proinflammatory and multifunctional molecule involved in innate immune responses against microbes, as well as in conditions of tissue injury associated with infections and sterile conditions. IL-1 is part of a large system, the IL-1 system, comprising a family of ligands that act as agonists, receptor antagonists, and anti-inflammatory cytokines, as well as a family of receptors that includes signaling receptor complexes, decoy receptors and negative regulators. All the members of the IL-1 system are involved in inflammatory diseases, innate and adaptive immune responses, conditions associated with dysmetabolism, and cancer by affecting both the tumor microenvironment and cancer cells. The deregulated or excessive activation of several pathways associated with the IL-1 system may lead to detrimental inflammatory or immune reactions, including autoinflammatory, autoimmune, infectious and degenerative diseases. The negative regulation of the IL-1 system mediated by antagonists, decoy receptors, scavengers, and dominant-negative molecules plays nonredundant roles in controlling these conditions. Owing to the central role of IL-1 in the pathogenesis of inflammation-driven diseases, IL-1 blocking agents are approved for clinical use in several inflammatory conditions, and inhibitory agents for other members of the family are under development. Here, the complexity of the IL-1 system, the involvement of its different members in inflammation-driven diseases, and the therapeutic approaches to target members of pathways associated with these conditions are presented and discussed.

## Introduction

Interleukin-1 (IL-1) is the first interleukin identified in the 1980s and is characterized as a potent proinflammatory molecule that contributes to innate immune responses against microbes and is involved in tissue injury. On the basis of sequence homology and genetic studies, several other IL-1-related ligands and receptors have been subsequently identified and collectively called the IL-1 system. Currently, the IL-1 system includes large and complex families of ligands and receptors involved in host responses in infections and inflammation, as well as in the activation of innate and adaptive lymphoid cells.

**Fig. 1:**
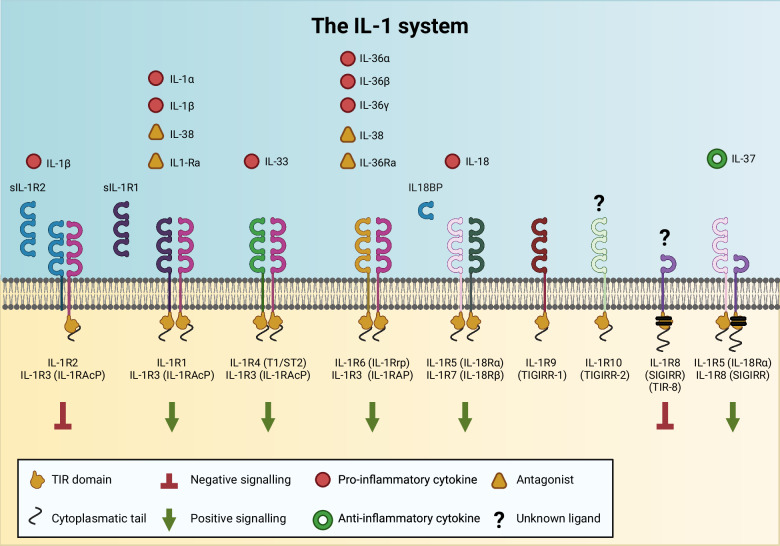
Overview of the interleukin-1 (IL-1) family system. The IL-1 system comprises seven agonists (IL-1α, IL-1β, IL-18, IL-33, IL-36α, β, and γ), an anti-inflammatory cytokine (IL-37) and eleven receptor molecules (IL-1R1-IL-1R10). Five functional signaling complexes are formed by pairing primary receptors with coreceptors. The system is tightly regulated by negative modulators, including 3 receptor antagonists (IL-1Ra, IL-36Ra, and IL-38), two decoy receptors, IL-1R2 and IL-18 binding protein (IL-18BP), and the inhibitory receptor IL-1R8

IL-1 family ligands include 7 agonists (IL-1α, IL-1β, IL-18, IL-33, IL-36α, β, and γ), 3 receptor antagonists (IL-1Ra, IL-36Ra, and IL-38), and an anti-inflammatory cytokine (IL-37) (Fig. 1). The IL-1 receptor (ILR) family members include 11 molecules [IL-1R1, IL-1R2, IL-1R3 (IL-1RAcP), IL-1R4 (ST2), IL-1R5 (IL-18Rα), IL-1R6 (IL-1Rrp2, IL-36R), IL-1R7 (IL-18Rβ), IL-1R8 (also known as SIGIRR and TIR8), IL-1R9 (TIGIRR-2), and IL-1R10 (TIGIRR-1)] (the new nomenclature based on sequential numerical order and previously used names, reported in brackets, are used in the literature and here) (Fig. 1) [1, 2]. The structure of receptor molecules includes an extracellular portion consisting of three Ig-like domains and an intracellular TIR domain (originally an acronym for Toll/IL-1 resistance and now for the Toll/IL-1R domain), which is essential for signaling via the MyD88 adapter and is shared by Toll-like receptors (TLRs). Upon interaction with ligands (IL-1 family cytokines, microbial ligands or selected danger signals), the TIR domain activates a conserved signaling cascade, leading to NF-κB translocation to the nucleus and the activation of mitogen‑activated protein kinases (MAPKs), such as p38, c‑Jun N‑terminal kinases (JNKs) and extracellular signal‑regulated kinases (ERKs) [3], leading to the amplification of innate immunity and inflammation.

Five signaling receptor complexes are formed: the IL-1 receptor [IL-1R1 and IL-1R3 (IL-1RAcP)], the IL-33 receptor [IL-1R4 (ST2) and IL-1R3 (IL-1RAcP)], the IL-18 receptor [IL-1R5 (IL-18Rα) and IL-1R7 (IL-18Rβ)], the IL-36 receptor [IL-1R6 (IL-1Rrp2 or IL-36R) and IL-1R3 (IL-1RAcP)], and the IL-37 receptor [IL-1R5 (IL-18Rα) and IL-1R8 (SIGIRR or TIR8)].

The IL-1 system includes negative regulatory molecules and anti-inflammatory cytokines that maintain the balance between the amplification of innate and adaptive immunity and uncontrolled inflammation. Indeed, deregulated or excessive activation of the IL-1 system is involved in the pathogenesis of severe and detrimental local or systemic inflammatory reactions and autoimmune or allergic responses, which are involved in many conditions, ranging from infections and acute tissue injury to cardiovascular diseases and cancer.

IL-1 blocking agents are approved for clinical use in several inflammatory conditions and have a tremendous impact on these diseases, and inhibitory agents for other members of the family are under development.

### IL-1

In humans, the *IL1A* and *IL1B* genes are positioned in close proximity on chromosome 2. Both IL-1α and IL-1β are transcribed and translated as precursor proteins, which do not contain a classical signal peptide and require proteolytic processing to generate the mature form, corresponding to the C-terminal region of the precursors.

Although IL-1α and IL-1β bind to the same membrane receptor, IL-1R1, they differ markedly in their cellular expression, regulatory mechanisms, and biological roles. IL-1β is produced mainly by monocytes, macrophages, dendritic cells (DCs), B cells, neutrophils, and natural killer (NK) cells, typically in response to stimulation by microbial products via TLRs, complement proteins, or other cytokines. In contrast, IL-1α is constitutively expressed in nonimmune cells, including endothelial cells, keratinocytes, fibroblasts, platelets, epithelial cells, and astrocytes. Myeloid cells are also capable of producing IL-1α, although this typically requires external stimulation.

The maturation and release of IL-1β require the activation of the inactive precursor procaspase-1 to caspase-1 by the NLRP3 inflammasome [4]. When stimulated by inflammasome activators such as ATP, nigericin, serum amyloid A, or pneumolysin from *Streptococcus pneumoniae, neutrophils secrete large quantities of active IL-1β* [5, 6]. The caspase-1-mediated activation of pro-IL-1β to bioactive IL-1β can occur either in the cytoplasm or within secretory lysosomes. Once activated, active IL-1β exits cells through various pathways, including exocytosis of lysosomes, extrusion of plasma membrane microvesicles, and export via transporters or multivescicular bodies containing exosomes [7]. Moreover, caspase-1 cleaves gasdermin D, generating N-terminal gasdermin fragments, which oligomerize and create membrane pores, enabling the release of mature IL-1β from the cell [8]. Finally, the IL-1β precursor can be liberated into the extracellular space by dying cells and converted into the active form by various proteolytic enzymes extracellularly. These include neutrophil proteinase-3, matrix metalloproteinase 9 (MMP9), elastase, granzyme B, and mast cell chymase [9].

Unlike the IL-1β precursor, the IL-1α precursor is biologically active and is released into the extracellular space following necrotic cell death, where it acts as an “alarmin,” initiating sterile inflammation [10]. Its proinflammatory potential can be further amplified through cleavage by proteases such as neutrophil elastase, chymase and thrombin [9, 11]. Another distinctive feature of IL-1α is its ability, when glycosylated, to associate with the plasma membrane of certain cells via a lectin-like interaction, enabling it to signal to neighboring cells in a juxtacrine fashion.

**Fig. 2:**
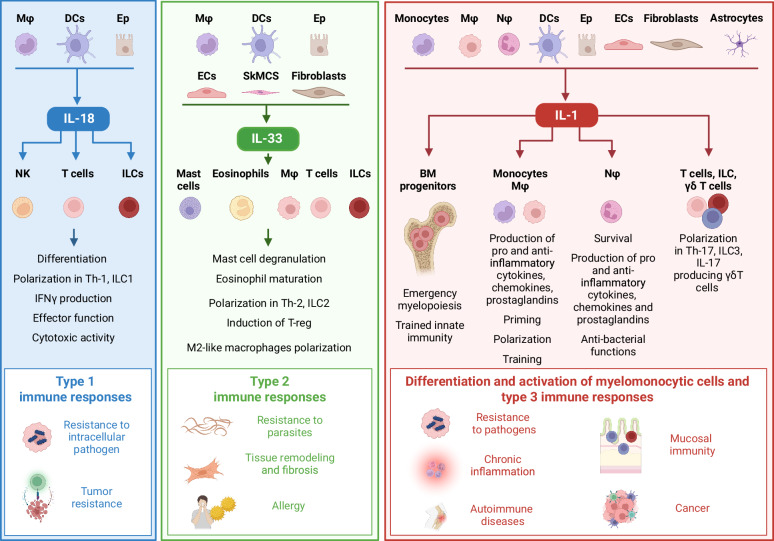
IL-1 system in innate and adaptive immune responses. Schematic representation of type 1, type 2 and type 3 immune responses activated by IL-18, IL-33 and IL-1, respectively

Activation of the IL-1R complex initiates a signaling cascade that drives the expression of a broad set of genes involved in inflammation, including cytokines, chemokines, and adhesion molecules, contributing to the amplification of the inflammatory response (Fig. 2 and see below). Moreover, IL-1 exerts various systemic effects; it acts as an endogenous pyrogen, inducing fever, which facilitates leukocyte migration, and activates the hypothalamic‒pituitary‒adrenal axis, ultimately promoting cortisol release to modulate inflammation. IL-1 promotes the production of IL-6, which triggers the acute phase response in the liver, enhances the humoral arm of innate immunity through the induction of proteins such as C-reactive protein and complement components, and contributes to the control of tissue damage through molecules such as α1-antitrypsin [12].

IL-1α has distinctive features. In the N-terminal pro-domain, known as the pro-piece, a nuclear localization signal enables pro-IL-1α to translocate into the nucleus and modulate gene expression through interactions with chromatin, transcription factors, and other regulatory proteins. This peculiar feature positions IL-1α, alongside IL-33 and IL-37, in the category of ‘dual-function’ cytokines, which are capable of both associating with nuclear chromatin and binding to surface receptors. During apoptosis, cytosolic IL-1α relocates to the nucleus, where it binds tightly to chromatin, limiting its ability to trigger an inflammatory response. Conversely, during necrosis, IL-1α is released into the cytosol, where it becomes available for extracellular release and the initiation of inflammation. Proteolytic cleavage by enzymes such as calpain, granzyme B from NK cells, or neutrophil elastase removes the nuclear localization domain, producing a mature form of IL-1α that cannot be longer retained in the nucleus and is available for secretion [1].

### IL-18

Unlike IL-1β, the precursor of IL-18 is constitutively expressed in various cell types, including monocytes, macrophages, and endothelial cells [13]. Furthermore, high amounts of pro-IL-18 are stored in epithelial barrier tissues such as the skin, lungs, and entire gastrointestinal tract [14]. Upon activation by caspase-1, the precursor is cleaved into mature IL-18 and secreted, which plays a major role as a proinflammatory mediator. In addition, the IL-18 precursor can be released from dying cells and cleaved to its active form extracellularly by proteases such as proteinase-3. As described for IL-1*α*, IL-18 can associate with the cell membrane, especially in macrophages differentiated with M-CSF [15].

IL-18 initiates a signaling cascade by binding to the IL-18Rα/IL-18Rβ receptor complex, ultimately leading to NF-κB nuclear translocation. Structurally, both the IL-18 precursor gene and its encoding gene are significantly similar to those of IL-37, which also utilizes IL-18Rα, suggesting a close relationship between these cytokines. Functionally, IL-18 is described mainly as a potent inducer of the type-1 immune response and as a potent IFNγ-inducing factor [15–17] (Fig. 2). The activity of IL-18 is controlled by IL-18 binding protein (IL-18BP), which functions as a decoy receptor, preventing IL-18 from engaging with the signaling receptor (see below).

### IL-33

IL-33 is a 30 kDa protein composed of an N-terminal domain containing a chromatin-binding motif that is responsible for the role of IL-33 as a chromatin-associated factor and an 18 kDa C-terminal region with cytokine-like activity and a structure similar to that of IL-1. A protease sensor linking the two domains contains cleavage sites for several proteases, including neutrophil and mast cell proteases (cathepsin G and chymase or tryptase, respectively), or allergen proteases, which release mature IL-33, leading to type 2 immunity and inflammation.

IL-33 is expressed in a constitutive manner in several stromal and parenchymal cell types associated with the cell nucleus. In particular, endothelial cells, mucosal epithelial cells, keratinocytes, fibroblasts, and fibroblastic reticular cells are major constitutive sources of IL-33 in several organs, with differences in terms of expression regulation between humans and mice. Localization in the nucleus is essential for regulating IL-33 release in the serum and subsequent type 2 immune responses, including eosinophilia, as well as systemic inflammation [18]. In contrast, nuclear localization is not associated with the regulation of transcription or cell-autonomous effects in IL-33-expressing cells [19], although there are discrepancies among studies. IL-33 constitutively stored in the nucleus may be released by unconventional mechanisms after injury, necrotic cell death or pyroptosis, infection by parasites or viruses, exposure to allergens, or cytokines, where it acts as an alarmin and causes sterile inflammation similar to that caused by IL-1α. In contrast, apoptosis-associated caspases 3 and 7 destroy IL-33. Upon release, the full-length 30 kDa protein is active; however, similar to other IL-1 family members, calpain and the neutrophil serine protease cathepsin G and elastase extracellularly cleave IL-33 and generate hyperactive mature forms [20].

Mature IL-33 signals through the IL-1R4 (ST2) receptor, which couples with the accessory protein IL-1R3 (IL-1AcP) to induce MyD88-dependent signaling and the activation of several MAPKs and NF-κB. ST2 is expressed on various innate and adaptive immune cell types involved in type 2 immune responses, particularly T helper-2 (Th2) cells, innate lymphoid cell-2 (ILC2) cells, type 2 CD8^+^ cytotoxic T cells, mast cells, eosinophils, basophils, DCs and alternatively activated M2-polarized macrophages. ST2/IL-1AcP signaling leads to the expression of type 2 cytokines (e.g., IL-5, IL-13, and IL-4), which are responsible for the activation of protective type 2 inflammatory responses during infection and tissue repair, as well as detrimental allergic responses, chronic obstructive pulmonary disease (COPD), and fibrosis [21, 22] (Fig. 2). IL-33 is also bound by the soluble forms of ST2 and IL-1RAcP, which are produced in inflammatory conditions or during trauma, resulting in the prevention of IL-33-dependent inflammation and type 2 immunity. This represents an important negative regulatory mechanism of IL-33 activity. In addition, IL-33 signaling activity is tuned by IL-1R8, which inhibits ST2 and IL-1RAcP dimerization (see below), and by the ubiquitin–proteasome system, which degrades ST2.

By inducing type 2 cytokines, IL-33 promotes eosinophil maturation and eosinophilia, playing a key role in parasitic infections and allergic reactions (see below). In addition, IL-33 induces the activation of signaling pathways associated with glucose and lipid metabolism, including peroxisome proliferator-activated receptor-γ (PPAR-γ), mammalian target of rapamycin (mTOR), and hypoxia-inducible factor 1α (HIF1α), promoting type 2 lymphoid cell activities [23]. In macrophages, IL-33 induces metabolic reprogramming that affects the mitochondrial respiratory chain, leading to increased production of itaconate and expression of the transcription factor GATA3. This pathway promotes the functional activity and skewing of M2-like macrophages, the resolution of inflammation and tissue repair [24]. ST2 is expressed by Tregs, including those located in nonlymphoid tissues, such as the intestine. In these cells, IL-33 promotes proliferation, regulatory functions and tissue repair by inducing the expression of IL-13 and amphiregulin [25].

IL-33 also promotes innate immunity and inflammatory responses. For example, IL-33 amplifies LPS-dependent cytokine and chemokine production in macrophages, increases myelopoiesis and neutrophil recruitment by inhibiting CXCR2 downregulation [26], upregulates CR3 expression in neutrophils, thus increasing *Candida albicans* phagocytosis and killing [27], and contributes to inflammation-associated pain by priming nociceptive neurons [28]. IL-33-driven type 2 immunity is generally associated with tumor promotion in both preclinical studies and human prognostic and genetic association studies. IL-33 enhances immune evasion by sustaining Treg-mediated immunosuppression through epigenetic reprogramming and by promoting M2-like macrophage polarization along with TGF-β production [29–32]. However, IL-33 can also support antitumor immunity by activating dendritic cells and boosting adaptive immune responses [33, 34].

Collectively, these studies show that by directly or indirectly affecting innate and adaptive lymphoid cells (ILC2 and Th2 cells, as well as Treg cells) and myeloid cells, including macrophages and mast cells, IL-33 is involved in several pathological conditions associated with type 2 responses, including allergies, infections, inflammation and cancer.

### IL-36

The IL-36 family includes IL-36α (IL-1F6), IL-36β (IL-1F8), and IL-36γ (IL-1F9), as well as IL-36Ra (IL-1F5), which act as receptor antagonists. IL-36-encoding genes are located on chromosome 2 in a cluster containing the genes encoding IL-1 and IL-1Ra. IL-36 family members are produced as precursors and acquire their complete functional activity upon cleavage of their N-terminus at the level of the A-X-Asp motif conserved in all IL-1 family members, which is mediated by proteases derived from neutrophils or epithelial cells, including cathepsin G, elastase, proteinase-3, and cathepsin S, as well as from bacteria and fungi [35]. Keratinocytes, bronchial epithelium, glial cells, monocytes/macrophages, T lymphocytes and γδT cells are the main cellular sources of IL-36 after stimulation with proinflammatory stimuli, e.g., TNFα, IL-17A, IL-22, IL-1β, and microbial components.

The IL-36 receptor, constituted by IL-1R6 (IL-1Rrp2) and IL-1R3 (IL-1RAcP) [36], activates a signaling pathway leading to NF-κB, MAPK, Erk1/2, and JNK activation and the expression of inflammatory cytokines, chemokines, and costimulatory molecules. IL-36Ra acts as a receptor antagonist, similar to IL-1Ra, by binding to IL-36R and blocking the recruitment of IL-1RAcP. IL-1Rrp2 is expressed by several cell types, particularly keratinocytes, fibroblasts, endothelial cells and leukocytes, under basal conditions or after inflammatory stimulation.

IL-36 cytokines are produced at high levels by epithelial cells of the skin and lung and contribute to innate immunity and acute or chronic inflammation in these tissues. In particular, IL-36 cytokines are involved in psoriasis and other neutrophilic and inflammatory skin disorders, such as atopic dermatitis, hidradenitis suppurativa, and autoimmune blistering disease [37], which depend on IL-36-induced IL-23, IL-17 and IL-22. Similarly, IL36Ra gene polymorphisms or deficiency of IL-36Ra (DITRA), a rare autosomal recessive autoinflammatory disorder, are associated with psoriasiform dermatitis [38]. In addition to skin diseases, aberrant activation of the IL-36/IL-36R axis has been described in inflammatory bowel disease, rheumatoid and psoriatic arthritis, and lung inflammation [39, 40]. By inducing IL-22, IL-36 promotes the resolution of intestinal mucosal inflammation and mucosal wound healing. However, in chronic intestinal inflammation, the IL‑36-dependent activation of fibroblasts contributes to intestinal inflammation progression and fibrosis [41].

IL-36γ also contributes to monocyte-trained immunity (see below) via NF-κB and mTOR-dependent epigenetic histone modifications, leading to increased cytokine responses and metabolic activity. This process is triggered by IL-36Ra or IL-38 (see below), which compete for IL-1R6 [42].

Thus, IL-36 cytokines share several properties with IL-1 but act as unique amplifiers of innate immunity and inflammation in select tissues, such as the skin, intestine and lung.

### IL-37

Five splice variants of IL-37 (IL-1F7) have been reported, of which IL-37b is the most complete and studied isoform. The IL-37 gene has been identified in humans but not in mice, although recombinant human IL-37 and transgenic human IL-37b are functional in mice [43].

IL-37 expression is induced by inflammatory stimuli (e.g., IL-1β or TLR ligands), as well as by the anti-inflammatory cytokine TGF-β [43]. IL-37 signals through IL-18Rα associated with IL-1R8, activating anti-inflammatory responses and inhibiting inflammatory cytokine production in innate immune cells [44–46]. In addition, IL-37 is associated with the nucleus and acts as a transcription regulator via colocalization with SMAD3, an anti-inflammatory kinase activated by TGF-β [43]. Moreover, IL-37a acts as a nuclear factor via IL-1R8-independent mechanisms by increasing PPAR-γ expression [47]. Among its functional activities, IL-37 inhibits DC-dependent T-cell responses, induces Tregs, and suppresses NK cell functions via IL-1R8, thus acting as an inhibitor of innate and adaptive immunity [45, 48, 49]. Preclinical studies in transgenic mice expressing the full-length IL-37b isoform (IL-37Tg) or treatment with recombinant IL-37 have shown that this cytokine plays anti-inflammatory roles, in most cases in an IL-1R8-dependent manner, under several infectious or sterile conditions, including LPS challenge, pulmonary aspergillosis, colitis, heart ischemia, liver injury, obesity-induced inflammation and insulin resistance, allergic airway inflammation, and arthritis [45, 50–52].

The *IL37* gene is located in close proximity to the *IL1B* gene, within the same topologically associating domain, a feature underlying their reciprocal regulation by *AMANZI* (a MAster noncoding RNA antagonizing inflammation), a long noncoding RNA cotranscribed with IL1B under inflammatory conditions and activating *IL37* expression. In turn, IL-37 negatively regulates *IL-1B* transcription, thus dampening inflammation in a cell-autonomous manner [53].

Genetic studies in humans support evidence from preclinical studies. For example, a homozygous *IL37* loss-of-function mutation has been associated with infantile inflammatory bowel disease [54]^,^ and *IL37* genetic variants are linked to impaired trained immunity [55].

### IL-38

The *IL38* gene is located in the chromosomal region containing the genes encoding for IL-1Ra and IL-36Ra. The IL-38 protein, which has primarily anti-inflammatory activity, shares high homology with IL-1Ra and IL-36Ra (>40%), and its structure is similar to that of IL-1Ra, suggesting an evolutionary link between these regulatory molecules. Basal epithelial cells of the skin and proliferating B cells of the tonsils are the major IL-38-producing cells. The IL-38 precursor is 152 amino acids long and has no signal peptide or caspase-1 consensus cleavage site. The IL-38 precursor has proinflammatory activity and promotes the production of proinflammatory cytokines, whereas the mature molecule, which is cleaved at its N-terminus, for example, in apoptotic cells, has anti-inflammatory biological activity.

IL-38 has been reported to interact with three different IL-1R family members, e.g., IL-1R6 (IL-1Rrp2), IL-1R1, and IL-1R9 (IL1RAPL1). By interacting with IL-1R6 (IL-1Rrp2), IL-38 may act as a partial receptor antagonist of IL-36R [56], whereas it reduces downstream signaling pathways upon interaction with the other two candidate receptors, IL-1R1 and IL-1R9 (IL1RAPL1) [57]. For example, upon stimulation with *C. albicans*, IL-38 negatively regulates IL-17 and IL-22 production by human memory T cells, although less effectively than does IL-1Ra [56]. A crystallized IL-38 signaling complex has not yet been reported. However, on the basis of the sequence of IL-38 and the finding that the anti-inflammatory activity of IL-38 (Th17 inhibition) is not dose dependent [56], it has been hypothesized that IL-38 is not a receptor antagonist of IL-36R but rather acts by recruiting a signaling receptor and functioning as an anti-inflammatory cytokine, similar to IL-37 [57].

Preclinical animal studies in inflammatory, autoimmune or metabolic disease models indicate that the main functional role of IL-38 is the negative regulation of inflammation. In agreement with these findings, genetic association studies in humans support the view that IL-38 affects inflammatory diseases, including metabolic syndrome, psoriatic arthritis and ankylosing spondylitis [1, 57]. In addition, IL-38 was shown to inhibit antitumor processes and sustain immunosuppression in preclinical cancer models. Indeed, antibody-mediated targeting of IL-38 reactivates immunostimulatory mechanisms in the tumor microenvironment and has anticancer effects [58].

Acute inflammatory conditions in patients are associated with increased concentrations of plasma IL-38, which are negatively correlated with the severity of the disease, suggesting that IL-38 induction reflects the activation of a negative feedback loop in these conditions. Increased IL-38 plasma concentrations have been reported in sepsis, autoimmune diseases, COPD and acute lung disorders, such as influenza and COVID-19, metabolic diseases and myocardial infarction [57, 59, 60].

Collectively, these studies indicate that IL-38 expression and function under inflammatory conditions reflect a regulatory loop that limits inflammation, including conditions associated with exacerbated trained immunity, but may also lead to detrimental immune suppression, which affects immune defenses during infection or cancer.

## Negative regulation of the IL-1 system

The negative regulation of the IL-1 system plays a fundamental role in tuning potentially detrimental inflammatory responses and promoting the resolution of the response. Regulation occurs at multiple levels and by diverse mechanisms, including ligands with receptor antagonist activity or anti-inflammatory functions, decoy receptors, negative regulatory receptors, and soluble forms of signaling receptors or accessory proteins (Fig. 1).

IL-1Ra was the first identified and main receptor antagonist of the IL-1 system. It binds IL-1R1 with higher affinity than does IL-1 and does not recruit IL-1RAcP. IL-1Ra is a secreted molecule, and two intracellular isoforms constitute a reservoir of IL-1Ra, which is released upon cell death. IL-1Ra deficiency in mice leads to spontaneous and lethal arteritis, destructive arthritis, psoriatic-like skin lesions, and increased susceptibility to carcinogenesis [1]. In humans, IL-1Ra genetic deficiency (DIRA) or loss-of-function mutations are associated with severe systemic and local inflammation, including pustular skin eruptions, vasculitis, osteolytic lesions and sterile osteomyelitis [61, 62]. The recombinant form of IL-1Ra (Anakinra) is used in clinics to treat different inflammatory conditions, including rheumatoid arthritis and autoinflammatory diseases such as cryopyrin-associated periodic syndrome (CAPS), and is currently in several clinical trials (see below).

IL-36Ra is the second receptor antagonist of the family, acting on IL-36R and inhibiting the production of cytokines induced by IL-36, such as IL-23, IL-17 and IL-22. IL-36Ra is a potential drug for psoriasiform dermatitis. Similar to IL-1Ra genetic deficiency, mutations in the IL-36Ra gene, collectively known as DITRA, are associated with life-threatening inflammatory conditions, the main clinical feature of which is severe psoriasis [38].

IL-1R2 was the first decoy receptor identified. In its cell-associated form, IL-1R2 binds to IL-1 with high affinity but does not signal since it lacks a TIR domain [63]; it also sequesters IL-1RAcP, acting as a dominant-negative regulator and reducing its availability for the signaling complex. In addition, in their soluble form, IL-1R2 and IL-1RAcP bind to pro-IL-1β with high affinity, blocking its processing by caspase-1 [1]. Finally, an intracellular form has been shown to interact with pro-IL-1α, preventing cleavage and activation by different enzymes (calpain, granzyme B, chymase, and elastase) during necrosis [64]. IL-1R2 expression is limited to a few cell types, particularly myeloid cells (neutrophils, monocytes, polarized M2 macrophages, and microglia), B cells and T regulatory cells, in contrast with IL-1R1, which is broadly expressed. IL-1R2 expression is upregulated by anti-inflammatory signals, including glucocorticoids, prostaglandins, and Th2 cell-associated cytokines, whereas proinflammatory stimuli (IFNγ, LPS and TNF) downregulate IL-1R2 protein levels [2, 7, 63]. Tumor-infiltrating Tregs express high levels of IL-1R2 associated with other immune checkpoint molecules, as shown by single-cell RNA-seq analysis of colorectal cancer, non-small cell lung cancer and breast carcinoma samples [65–67]. These results suggest that IL-1R2 might be a target to inhibit Treg-mediated suppression, as suggested by a study focused on the cross-talk between IL-1R2^+^ Tregs and cancer-associated fibroblasts [68]. IL-1R2 upregulation and release in soluble form are observed in several inflammatory diseases in patients, including critically ill patients with sepsis, acute meningococcal infection, trauma, necrotizing enterocolitis in preterm infants, and acute respiratory distress syndrome, such as COVID-19, and are correlated with the severity of the disease [69–72]. In sepsis patients, IL-1R2 is associated with a cluster of monocytes characterized by low levels of HLA-DR and limited TNF production [69], as well as by the coexpression of mature macrophages and immune dysfunction features, which correlate with immunological markers and clinical parameters (e.g., SOFA score, creatinine, survival), reflecting infection severity [73]. Dexamethasone treatment of COVID-19 patients was associated with the upregulation of IL-1R2 in immunosuppressive neutrophils.

The second decoy molecule of the system is IL-18BP, which functions as a soluble, high-affinity decoy for IL-18 and is present in the circulation at 20-fold higher molar concentrations than does IL-18. IL-18BP limits IL-18 activity by negatively regulating the IL-18-dependent induction of IFNγ and Th1 responses. IL-18BP may inhibit IL-18 immunotherapy, acting as a soluble immune checkpoint [74]. An imbalance of IL-18 and IL-18BP results in elevated free IL-18 circulating levels and contributes to IL-18-mediated diseases associated with inflammasome hyperactivation or pyroptosis, such as autoinflammatory diseases and macrophage activation syndrome [75, 76].

IL-1R8 (also known as TIR8 or SIGIRR) is a receptor characterized by a single Ig domain and a nonconventional TIR domain that negatively regulates the signaling pathway activated by most IL-1R family members or TLRs. It is highly expressed in the epithelial cells of several organs, as well as in lymphoid organs [77]. Its mode of action includes interference with the recruitment of TIR-containing adaptor molecules and prevention of the translocation of the signalosome from the receptor, thus limiting NF-κB and JNK activation [78, 79]. In addition, IL-1R8 affects mTOR kinase activity in Th17 lymphocytes [80] and in intestinal epithelial cells [81]. Finally, IL-1R8 acts as a receptor chain for IL-37, together with IL-18Rα, and is essential for the anti-inflammatory activities of IL-37, as discussed above.

IL-1R8 plays nonredundant regulatory roles in controlling detrimental inflammation and immune responses in infections (e.g., tuberculosis, candidiasis, *P. aeruginosa* infection) [82–84], colitis [78, 85], kidney ischemia or transplantation [86, 87], neuroinflammation and cognitive impairment [88]. IL-1R8 negatively regulates cancer-related inflammation in intestinal carcinogenesis [81, 89], chronic lymphocytic leukemia (CLL) [90], and diffuse-large B-cell lymphoma [91]. In specific infections, such as urinary tract infection (UTI) caused by uropathogenic *E. coli* or pneumonia and sepsis caused by *S. pneumoniae*, the regulatory role of IL-1R8 inhibits the development of protective innate and inflammatory responses [92, 93].

The negative regulatory activity mediated by IL-1R8 in lymphoid cells in response to IL-1 family members confers features of an immune checkpoint to this molecule. Indeed, IL-1R8 is involved in tuning IL-33-dependent Th2 responses in allergies [94], IL-1-induced Th17 activation in autoimmunity [80, 95], and IL-18-induced Th1 responses and NK cell antitumoral and antiviral functional activation [96]. In particular, IL-1R8 affects NK cell development and functions by regulating IL-18 signaling and IFNγ production, with a negative impact on the NK cell-mediated control of hepatocellular carcinoma, lung metastasis, colon cancer-derived liver metastasis, and cytomegalovirus (CMV) infection in preclinical models [96]. Silencing IL-1R8 in human NK cells improves their antitumor function [97], indicating that targeting IL-1R8 in NK cells is clinically relevant. An immunomodulatory role of IL-1R8 in human cancer has been proposed on the basis of the association between its expression by breast cancer cells and impaired innate immune sensing and T-cell responses [98].

## Regulation and orchestration of the immune response by IL-1 family members

### Effects of IL-1 on myeloid cells, trained innate immunity and emergency myelopoiesis

Myeloid cells, including monocytes, macrophages, and neutrophils, are well-known producers of various cytokines, including IL-1 family members. IL-1 can induce its own production both in vivo and in vitro in monocytes [7]. Monocytes and macrophages rely on caspase-1 activation for IL-1 processing, whereas neutrophils can process IL-1 through both inflammasome-dependent and inflammasome-independent mechanisms [7].

IL-1β is a critical modulator of the functional activity and survival of mature myeloid cells, as well as of the fate of hematopoietic progenitors in the bone marrow and extramedullary sites; thus, it plays a central role in inflammation, emergency myelopoiesis and trained innate immunity [2].

**Fig. 3:**
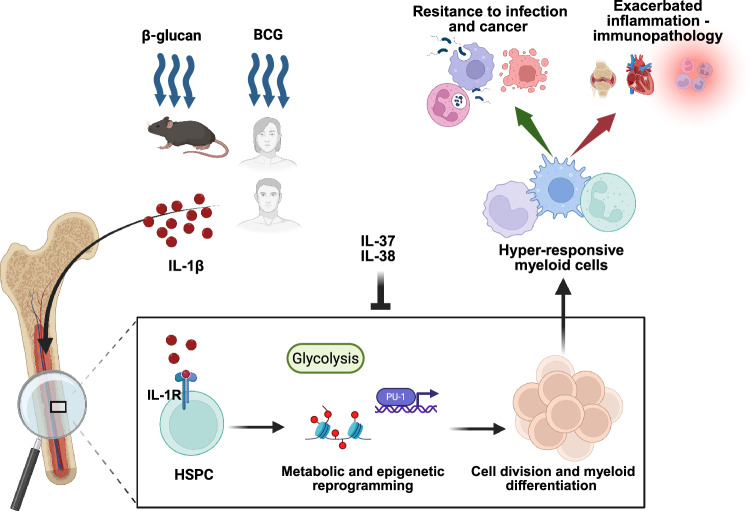
IL-1β-dependent induction of trained innate immunity. Exposure to microbe-derived components such as β-glucan or Bacillus Calmette-Guérin (BCG) triggers an inflammatory response characterized by the production of IL-1β. IL-1β acts on hematopoietic stem and progenitor cells (HSPCs) in the bone marrow via IL-1R signaling, leading to epigenetic and metabolic reprogramming, including increased glycolysis and activation of the PU.1-dependent myeloid gene program. This reprogramming promotes accelerated proliferation and differentiation of HSPCs into myeloid lineage cells. The resulting hyperresponsive myeloid cells exhibit enhanced effector functions upon secondary stimulation, such as increased cytokine production, improved pathogen phagocytosis and increased cytotoxic activity against tumor cells. While this trained immunity can confer protection against infections and malignancies, it can also contribute to chronic inflammation and immunopathology, such as arthritis, cardiovascular diseases and autoimmune diseases. Conversely, IL-37 and IL-38 have the capacity to inhibit IL-1β-dependent trained immunity

While immunological memory has traditionally been considered a hallmark of adaptive immunity, there is growing evidence for the concept of trained innate immunity. This phenomenon involves long-term functional reprogramming of innate immune cells, which enhances their responsiveness to subsequent challenges. Experimental studies in mice have demonstrated that priming with microbial ligands or sterile inflammation can train the innate immune system, providing protection against subsequent infections [99, 100]. Trained innate immunity is orchestrated by a combination of metabolic and epigenetic adaptations, resulting in local cell expansion, enhanced myelopoiesis, improved metabolism, and increased production of effector molecules, such as cytokines and reactive oxygen species, upon restimulation (Fig. 3). Several stimuli have been shown to induce trained innate immunity, including the malaria pathogen *Plasmodium falciparum*, the fungal cell wall component β-glucan, the metabolite fumarate, soluble uric acid, the Bacillus Calmette-Guérin (BCG) vaccine, oxidized low-density lipoprotein (oxLDL), and IL-1β [101–104]. Conversely, IL-37, which suppresses innate immune responses, reverses the immune-metabolic and epigenetic changes associated with trained immunity in monocytes, thereby eliminating the host defense and survival benefits conferred by trained innate immunity in vivo [105]. Similarly, IL-38 has emerged as a negative regulator of trained immunity. For example, in preclinical models of trained immunity induced by β-glucan, IL-38 negatively regulates mTOR signaling, thus preventing trained immunity-associated epigenetic and metabolic changes [42, 106].

Although the term “trained innate immunity” was first proposed in 2011 [107], protective activity against lethal bacterial and fungal infections following pretreatment with IL-1β was reported as early as 1984 [108]. In 2018, a study identified IL-1β as a key mediator in β-glucan-induced trained innate immunity in mice [104]. Administration of β-glucan led to the expansion of hematopoietic stem and progenitor cell (HSPC) populations and increased myelopoiesis, which was associated with increased glycolysis in HSPCs. Since HSPCs do not express the β-glucan receptor Dectin-1, their expansion and reprogramming must occur through indirect mechanisms. Pharmacological inhibition of IL-1β by IL-1Ra (anakinra) prevents increased myelopoiesis after the administration of β-glucan [104]. Mice treated with β-glucan showed significant protection against pulmonary *Mycobacterium tuberculosis* infection, which correlated with the expansion of HSPCs [109]. In line with the key role of IL-1 in this process, this protective effect was lost in IL-1R1-deficient mice. The β-glucan-induced expansion of HSPCs resembles an emergency myelopoiesis response, and IL-1β has been shown to promote the proliferation and myeloid differentiation of HSPCs. Under pathological conditions, including infection and cancer, high levels of IL-1 drive emergency hematopoiesis by directly accelerating the division and myeloid differentiation of hematopoietic stem cells, resulting in increased myeloid cell production to meet the increased demand [110]. In humans, BCG vaccination induces reprogramming in monocytes through an IL-1β-dependent mechanism, leading to increased cytokine production and protection against experimental viral infection [111]. Similarly, polymorphisms in genes encoding IL-1 family members, including IL-1β, influence the production of cytokines associated with trained immunity [111]. In addition, exposure of human HSPCs to IL-1β induces a shift toward myeloid cell proliferation, increased cytokine expression in HSPC-derived monocytes and increased phagocytosis by monocyte-derived macrophages [112].

In addition to its impact on HSPCs, IL-1β can induce trained immunity in mature cells, including monocytes and macrophages [111, 113]. Mechanistically, trained monocytes undergo a metabolic shift characterized by increased glycolysis dependent on the activation of the AKT/mTOR/HIF-1α signaling pathway [114]. Activation of the transcription factor HIF1α leads to the expression of its target genes, including IL-1β [115]. In turn, IL-1β itself can sustain HIF1α expression and activity, further promoting a trained phenotype in human monocytes through epigenetic reprogramming [111, 116].

### Orchestration of IL-1-mediated immune and inflammatory responses by neutrophils

Neutrophils are an unexpected source of cytokines, including members of the IL-1 family [117]. The pore-forming toxin pneumolysin from *S. pneumoniae* triggers the activation of the NLRP3 inflammasome in neutrophils, leading to caspase-1 activation and IL-1β secretion. This process highlights the role of neutrophils as the dominant source of IL-1β in murine models of infection by *S. pneumoniae* [6].

Recent transcriptomic studies, including bulk and single-cell RNA sequencing, have revealed the complexity and diversity of neutrophils under both steady-state and inflammatory conditions [118–120]. During neutrophil maturation, from immature preneutrophils primarily found in the bone marrow to fully mature neutrophils in the blood and spleen, IL-1β expression is predominantly observed in mature neutrophils [120]. Under steady-state conditions, neutrophils can acquire transcriptional signatures specific to the tissues they infiltrate. For example, lung neutrophils presented elevated IL-1β expression, which was associated with a proangiogenic transcriptional profile [120]. In humans, low-density neutrophils (LDNs), a heterogeneous population comprising neutrophil precursors, immature neutrophils, and mature neutrophils, exhibit lower basal levels of IL-1β than normal-density mature neutrophils (NDNs). However, compared with mature NDNs, LDNs show an increased capacity to produce IL-1β in response to TLR8 ligands [118]. The upregulation of IL-1β in inflammatory conditions, such as cancer and infection, is conserved in both human and murine neutrophils [121].

In addition to its antimicrobial functions, neutrophil-derived IL-1β contributes significantly to pathological inflammation. This phenomenon is evident in experimental models of inflammatory arthritis, cutaneous inflammation, colitis-associated tumorigenesis, autoinflammatory osteomyelitis, and endotoxin challenge in humans [122, 123]. Tumor-associated neutrophils have also been identified as a major source of IL-1β within the tumor microenvironment [124–126]. In a colitis model, neutrophil depletion led to a significant reduction in IL-1β expression in the colon, with neutrophil transfer being sufficient to restore IL-1β production, further emphasizing the critical role of neutrophils in IL-1β generation [127].

Additionally, neutrophils express IL-1Ra and the decoy receptor IL-1R2, which limits inflammatory responses and interferes with the senescence-associated secretory phenotype [63, 128]. Gene expression analysis of both mouse and human resting neutrophils revealed consistent expression of IL-1Ra and IL-1R2 [121]. In severe infections and sepsis, patient subsets with poor outcomes present increased numbers of circulating IL-1R2^+^ immature neutrophils, which possess immunosuppressive properties [129].

In addition to processing pro-IL-1β and pro-IL-18, activated caspase-1 can trigger pyroptosis, a form of lytic cell death that contributes to inflammation. Interestingly, unlike macrophages and dendritic cells, neutrophils are resistant to pyroptosis induced by *Salmonella typhimurium* both in vitro and in vivo [130]. This resistance allows neutrophils to act as a significant source of IL-1β during *Salmonella* infection, maintaining a unique inflammasome pathway that is resistant to caspase-1-driven pyroptosis. Neutrophils also express and release IL-18 upon exposure to inflammasome activators, such as *Legionella pneumophila*, thus contributing to activating NK cells to produce IFNγ and promoting the immune response [5].

### Polarization of innate and adaptive lymphoid cells

Members of the IL-1 family play pivotal roles in modulating immune responses by influencing the development, activation, and polarization of both innate and adaptive lymphoid cells, including T lymphocytes and ILCs [1, 131, 132] (see Fig. 2 and above).

ILCs constitute a heterogeneous group of lymphoid cells [132] categorized into three major subsets: group 1 ILCs, which include NK cells and other IFNγ-producing ILCs; group 2 ILCs, which are responsible for the production of type 2 cytokines (IL-4, IL-5, IL-9, and IL-13); and group 3 ILCs, which secrete IL-17 and/or IL-22. The differentiation and functional activity of these subsets are regulated by lymphoid-targeting cytokines, including γ chain (γc) cytokines (e.g., IL-2, IL-4, IL-7, IL-9, IL-15, and IL-21, which share the common cytokine receptor γc as a receptor component), and IL-1 family members. As already discussed above, IL-18, in conjunction with IL-15, acts as a key driver of IFNγ production in ILC1s; IL-33, in synergy with IL-25, promotes the expansion of ILC2s and the secretion of type 2 cytokines, including IL-5 and IL-13; and IL-1β, along with IL-23, activates ILC3s to produce IL-17 and IL-22. Similarly, IL-18 is crucial for the differentiation of Th1 cells and the induction of IFNγ secretion [133], whereas IL-12 facilitates Th1 responses by upregulating IL-18R expression. A distinct subset of Th1 cells, referred to as “nonclassical” Th1 cells due to their CD161 expression and developmental origin from Th17 cells, is characterized by IL-1R1 expression [134, 135]. IL-33 plays a central role in supporting Th2 responses and the production of type 2 cytokines, particularly IL-5 and IL-13 (see above).

IL-1 is a critical factor driving the differentiation of human naïve T cells into Th17 cells [136] and contributes to the development of Th17-driven autoimmune conditions [137]. Moreover, IL-1 acts as an IL-17-inducing factor in γδT cells and iNKT cells, particularly in response to infections [138, 139]. In addition to its role in T-cell differentiation, IL-1β enhances the effector functions of antigen-specific T cells and amplifies memory responses [140, 141].

Given the involvement of IL-1 family members in lymphoid cell polarization, their role in cancer immunology is particularly relevant. IL-18, by inducing IFNγ production in NK cells, ILC1s, and Th1 cells, exhibits antitumor activity. Both preclinical models and human cancer studies have linked IFNγ-related signatures, including IL-18, to improved prognosis [142]. Recent findings indicate that caspase-3-mediated cleavage of IL-18 in cancer cells generates a truncated, nonsecreted form of IL-18 that translocates into the nucleus, where it facilitates STAT1 phosphorylation. This process subsequently drives the expression and release of ISG15, promoting NK cell recruitment and supporting tumor suppression [143]. In contrast, IL-33-driven type 2 immunity is associated with tumor progression. Additionally, IL-1 promotes protumor myeloid bias and supports IL-17-dependent tumor progression in multiple solid malignancies, including skin, breast, prostate, and gastrointestinal cancers. This occurs through mechanisms that enhance tumor invasion, migration, angiogenesis, and resistance to chemotherapy [144–146].

## The role of IL-1 and IL-1R family members in immunopathology

### Infections

Sensing microbial moieties and tissue damage by pattern recognition molecules of the cellular arm of innate immunity activates a cytokine cascade that induces amplification and regulation of innate immunity. IL-1 is one of the primary inflammatory cytokines involved, together with IL-6 and TNF, by inducing the production of secondary mediators [e.g., IL-6 itself, chemokines, colony stimulating factors (CSFs), endothelial adhesion molecules, prostaglandins, nitric oxide (NO)], which amplify local and systemic innate immunity, such as leukocyte recruitment and effector functions, as well as emergency hemopoiesis. This cascade of events is sufficient to control infection in most cases and in all animal species that possess only innate immunity; however, in vertebrates, it drives the activation and orientation of adaptive immune responses against the pathogen.

In several infections, IL-1 plays nonredundant roles in this cascade, as indicated, for example, by increased susceptibility to *S. pneumoniae, Staphylococcus aureus*, *M. tuberculosis*, or fungal infections in IL-1- or IL-1R1-deficient mice (e.g., [147, 148]). In humans, *IL1* polymorphisms have been associated with susceptibility to *Helicobacter pylori, Neisseria meningitides, and M. tuberculosis*. For example, the *IL1B* rs16944 polymorphism is associated with susceptibility to different infections, including invasive fungal infections and tuberculosis [149, 150].

Balanced IL-1-dependent inflammation is essential to prevent detrimental inflammation and immunopathology, as evidenced, for example, in severe bacterial or viral pneumonia, where neutrophils driving lung injury are the predominant source of IL-1β, or in infection models in which key negative regulators of the IL-1 system, such as IL-1R8, are deficient. In human infectious diseases, such as viral respiratory diseases associated with acute respiratory distress syndrome (ARDS), IL-1 plays a major role in hyperinflammation. A key example is COVID-19, where NLRP3 inflammasome activation by SARS-CoV-2 results in massive release of IL-1β and IL-18, leading to cytokine release syndrome, with hyperactivation of myeloid cells and Th17 responses or IL-18-driven IFNγ production and Th1 responses. In agreement with these findings, several trials in patients hospitalized with moderate and severe COVID-19 revealed that early start of treatment with anakinra, guided by markers of inflammatory burden, significantly reduced the risk of progression to severe respiratory failure and mortality [151, 152].

Given the pivotal role of IL-1 family-driven inflammation in a wide range of pathologies, several IL-1-targeting therapies have been developed, including anakinra, canakinumab (anti-IL-1β mAb)), and rilonacept (a soluble IL-1 receptor chimeric fusion protein neutralizing IL-1α and IL-1β), which have received approval from both the United States Food and Drug Administration (FDA) and the European Medicines Association (EMA) for the treatment of various autoinflammatory and autoimmune conditions [153]. Anakinra is used to treat several inflammatory diseases, including rheumatoid arthritis, gout, and idiopathic pericarditis, as well as rare autoinflammatory diseases associated with deregulated IL-1 production (see below). In these patients, opportunistic infections due to anakinra are rare, including in populations at high risk for reactivation of *M. tuberculosis* infections, possibly because of the short half-life of the drug. In a large study, the Canakinumab Anti-inflammatory Thrombosis Outcomes Study (CANTOS), involving more than 10000 patients, reported that treatment with canakinumab for 4 years increased susceptibility to infections, including *M. tuberculosis* and sepsis [154].

In addition to other members of the IL-1 family, IL-33 is a key cytokine in parasite infections and drives the activation of ILC2 and Th2 cells and the induction of type 2 cytokines (in particular, IL-13), which promote intestinal nematode expulsion or control *Toxoplasma gondii* encephalitis [155, 156]. During fungal or viral infections, IL-33 sustains Treg cell suppressive functions, whereas IL-1 promotes the acquisition of a Th17 phenotype and antipathogen responses [157]. In sepsis, IL-33 released during tissue injury contributes to immunosuppression by activating ILC2s, which promote the polarization of M2-like macrophages, thereby enhancing the IL-10-dependent expansion of Tregs [158]. During viral infections, IL-33 promotes type 1 responses in CD4^+^ Th1 and CD8^+^ T cells, which transiently express ST2 upon antigen recognition and stimulation with IL-12, including the production of IFNγ and TNF [159]. In chronic viral infections, IL-33 is involved in preserving the stemness of T-cell factor 1 (Tcf-1)-expressing CD8^+^ T cells and their role in controlling the viral load by epigenetically regulating chromatin accessibility [160].

As a powerful inducer of type-1 responses and cytotoxicity in innate and adaptive lymphocytes, IL-18 plays prominent roles in infections, particularly those caused by intracellular bacteria, fungi and protozoa, as well as viruses. IL-18, including a membrane-bound form expressed on monocytes-macrophages, cooperates with STAT-inducing cytokines (e.g., IL-12 and/or IL-15) to activate NK and T-cell effector functions, such as IFNγ production, cytotoxicity and FasL expression, thus promoting resistance to microbial infections or the microbicidal activity of macrophages [15, 161]. On the other hand, this pathway may contribute to immunopathology and cytokine release syndrome in severe infections, including COVID-19 [162].

IL-36 cytokines promote DC activation as well as Th1 and Th17 cell polarization and proliferation, which are associated with mucosal immunity, particularly in infections caused by fungi (*C. albicans* and *Aspergillus fumigatus*) [163] or bacteria [164]. In viral infections, the role of the cytokine IL-36 is dual, depending on the condition and severity of the infection. They are associated with protective immune responses due to the induction of type 1 IFNs, inflammatory cytokines and chemokines; the promotion of immune cell activation and differentiation; mucosal integrity; and detrimental inflammatory cascades [165].

### Acute and chronic sterile inflammatory diseases

**Fig. 4:**
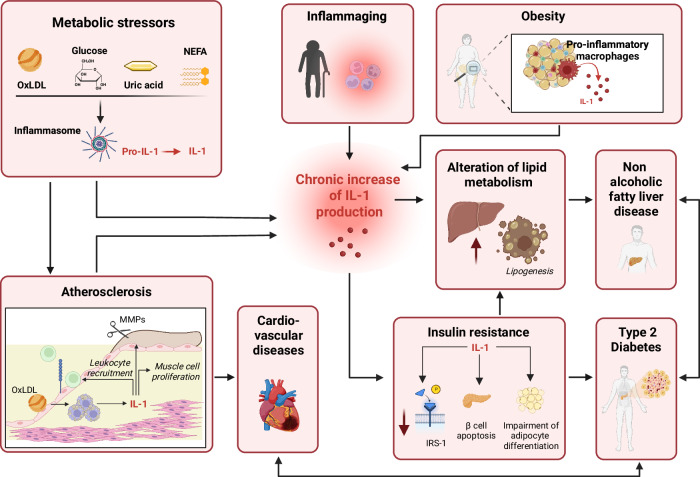
Persistent IL-1 production drives the pathogenesis of sterile chronic inflammatory diseases. Persistent IL-1 production sustains the vicious cycle between metabolic stress and chronic inflammation, driving the pathogenesis of chronic inflammatory diseases such as cardiovascular diseases, type 2 diabetes, and nonalcoholic fatty liver disease. Metabolic stressors activate the inflammasome, resulting in increased levels of IL-1 that perpetuate chronic inflammation. This mechanism is central to the generation, progression, and eventual rupture of atherosclerotic plaques. Oxidized low-density lipoprotein (OxLDL) and cholesterol crystals accumulate in macrophages within arteries, promoting foam cell formation and inflammasome activation. This results in IL-1 production, which in turn leads to the activation of endothelial cells, further recruitment of leukocytes, and vascular smooth muscle cell proliferation, inducing plaque growth. Additionally, it promotes the production of matrix metalloproteinases (MMPs), which, by weakening the fibrous cap of the plaque, increase the risk of plaque rupture and thrombosis. Ultimately, atherosclerosis predisposes patients to cardiovascular diseases. Inflammaging and obesity lead to a chronic increase in IL-1 production. Persistent IL-1 impairs glucose metabolism by disrupting insulin signaling, reducing insulin receptor substrate 1 (IRS-1) phosphorylation, inducing pancreatic β-apoptosis and impairing the differentiation of adipocytes. Through these mechanisms, chronic IL-1 elevation can promote insulin resistance and the development of type 2 diabetes. Chronically elevated IL-1 levels also induce hepatic lipogenesis, predisposing patients to nonalcoholic fatty liver disease (NAFLD). Insulin resistance exacerbates this condition by further stimulating lipid accumulation in the liver. Insulin resistance, type 2 diabetes, NAFLD, atherosclerosis, and cardiovascular diseases are closely related. Insulin resistance promotes vascular damage by inducing endothelial dysfunction and inflammation. Conversely, atherosclerosis increases systemic inflammation, which worsens glucose metabolism and further exacerbates insulin resistance. Moreover, insulin resistance-driven hepatic lipogenesis worsens NAFLD, with downstream effects on lipid metabolism and cardiovascular risk

In addition to their well-established roles in infections, cytokines of the IL-1 family are central mediators of acute and sterile inflammation. By responding to endogenous danger signals released during tissue damage or stress, they contribute to immune activation processes that play a central role in the pathogenesis of cardiovascular, metabolic, and neurodegenerative disorders [166, 167] (Fig. 4).

A clear example of acute sterile inflammation that involves IL-1 family members is myocardial infarction (MI). Upon myocardial infarction, massive tissue damage and cell death resulting from ischemia activate the NLRP3 inflammasome, leading to the production of IL-1β and IL-18, which further promote adverse remodeling, ultimately contributing to heart failure (HF) [168–171]. The concentrations of these cytokines increase in heart tissue and in the circulation of mice and humans upon MI [172]. In experimental models, IL-1R1-deficient mice show reduced myocardial damage and more favorable remodeling post-MI, whereas IL-1Ra-deficient mice experience exacerbated adverse remodeling, emphasizing the detrimental role of IL-1 signaling in this pathology [173, 174]. Consistently, treatment with IL-1Ra, anti-IL-1β antibodies and IL-1 Trap (an IL-1 antagonist) has shown protective effects in preclinical studies [175–178]. The results obtained in animal models are further corroborated by clinical trials. In the VCU-ART clinical study, treatment with anakinra reduced the incidence of new-onset HF and hospitalization for HF, whereas in the CANTOS trial, treatment with the IL-1β blocking antibody canakinumab significantly reduced the occurrence of recurrent cardiovascular events compared with a placebo [179–181]. IL-18 also has some clinical relevance for MI, as genetically predicted IL-18 inhibition is associated with favorable cardiovascular outcomes [182].

Ischemic stroke is another acute sterile inflammatory event in which IL-1 family cytokines play important roles. In ischemic stroke experimental models induced by middle cerebral artery occlusion (MCAo), IL-1α expression was found to be associated with the area of damage [183]. Mice deficient in both IL-1α and IL-1β exhibit significantly reduced tissue injury, whereas intraventricular administration of IL-1β-blocking antibodies or the IL-1β antagonist anakinra reduces infarct size and ischemic injury in experimental MCAos in rats [184, 185]. Notably, the use of IL-1Ra has been suggested to contribute to better clinical outcomes in stroke patients [186].

The role of IL-1 family members in sterile inflammation-mediated pathologies extends far beyond these acute events and is at the center of chronic inflammatory conditions that drive a broad spectrum of chronic diseases, including type 2 diabetes (T2D), nonalcoholic fatty liver disease (NAFLD), atherosclerosis, and neurodegenerative disorders (Fig. 4). In these contexts, the persistent inflammatory state characteristic of chronic diseases is often sustained by underlying metabolic disturbances, such as obesity, metabolic syndrome, and hypercholesterolemia, which serve as continuous sources of sterile inflammatory stimuli and significantly increase susceptibility to these chronic diseases. IL-1 family members play a central role in mediating this bidirectional crosstalk between inflammation and metabolic dysfunction. At the molecular level, metabolic stressors, including high glucose, nonesterified free fatty acids (NEFAs), cholesterol crystals, and uric acid, directly or indirectly activate the inflammasome, leading to caspase-1-dependent maturation and the release of the proinflammatory cytokines IL-1β and IL-18 [187–191]. In addition, fatty acids induce IL-1α production in an inflammasome-independent manner [192]. In turn, IL-1 family cytokines establish a self-sustaining inflammatory loop that further exacerbates metabolic dysfunction by disrupting both glucose and lipid homeostasis.

A prototypical example of this loop is the link between chronic IL-1-mediated inflammation and T2D development. Under physiological conditions, food intake induces a physiological increase in macrophage-derived IL-1β, which promotes postprandial insulin secretion [193]. Moreover, IL-1β contributes to the cephalic phase of insulin release by enhancing its parasympathetic activation before glucose intake [194]. Consistently, IL-1R1-deficient mice develop glucose intolerance faster than their wild-type littermates do and exhibit impaired insulin secretion upon a high-fat diet [195]. However, when IL-1β levels remain chronically elevated, as observed in obesity and metabolic syndrome, its effects become detrimental, ultimately contributing to the development of diabetes. Persistently high levels of IL-1α and IL-1β impair insulin signaling by reducing insulin receptor substrate 1 (IRS-1) phosphorylation and disrupting its downstream pathways, leading to insulin resistance [196–198]. Furthermore, IL-1 impairs proper adipocyte differentiation, facilitating the generation of inflamed adipose tissue [196, 199], and directly induces pancreatic β-apoptosis [200]. Consistently, treatment with anakinra ameliorated glycemia and the secretory function of β cells and reduced the expression of markers of systemic inflammation in patients with T2D [201]. In addition to alterations in glucose metabolism, chronically elevated IL‑1β levels also negatively affect lipid metabolism by directly promoting de novo lipogenesis in hepatocytes, leading to hepatic lipid accumulation [202, 203]. This imbalance contributes to the onset and progression of NAFLD and hepatic steatosis, which are conditions commonly associated with obesity and type 2 diabetes [202, 204, 205]. Notably, IL‑1Ra treatment in obese mice improved steatosis and downregulated hepatic lipogenic gene expression [202]

Other IL-1 family members have been implicated in metabolism regulation under healthy and pathological conditions. IL-18, for example, plays a significant role in maintaining metabolic balance. Mice lacking IL-18 or its receptor (IL-18Rα) exhibit excessive food intake due to impaired appetite regulation by the central nervous system, which results in obesity, insulin resistance, atherosclerosis, and T2D [206]. In addition, IL-18 directly regulates energy metabolism, modulating insulin sensitivity in white adipose tissue through IL-18R signaling and controlling thermogenesis in brown adipose tissue via the sodium-chloride cotransporter [207]. Similarly, IL-33 derived from pancreatic islets enhances β-cell insulin secretion, activating resident ILC2s, which in turn stimulate macrophages and DCs to produce retinoic acid, which supports insulin secretion [208]. Notably, this IL-33-ILC2-retinoic acid axis becomes dysfunctional in obesity and can be restored by exogenous IL-33 treatment [208]. In contrast, the anti-inflammatory agent IL-37 protects against obesity-associated insulin resistance by attenuating adipose tissue inflammation and increasing adiponectin levels in animal models [51].

Chronic IL-1-mediated inflammation also plays a pivotal role in the development and progression of atherosclerosis, a major contributor to cardiovascular disease. Under these conditions, hyperlipidemia and impaired clearance of lipoproteins from the arterial wall lead to the formation of immune cell-rich plaques, where IL-1α, IL-1β, and IL-18 are abundantly expressed, especially those produced by infiltrating macrophages [209–213]. Atherogenic stimuli, including lipids, oxidized LDL (oxLDL), cholesterol crystals, products of lipid peroxidation, and damage-associated signals released by dying cells in the necrotic core of plaques, are able to directly induce the NLRP3 inflammasome [190, 212, 214, 215]. Once activated, IL-1 promotes endothelial cell activation and the upregulation of adhesion molecules, facilitating leukocyte recruitment to plaques. Additionally, it stimulates vascular smooth muscle cell proliferation and migration, processes that contribute to plaque growth and instability. Furthermore, IL-1β enhances the expression of matrix metalloproteinases (MMPs), which degrade the fibrous cap of the plaque and contribute to its rupture, eventually resulting in thrombosis [216, 217].

The pro-atherogenic role of IL-1 and IL-18 has been clearly demonstrated in experimental models in which mice lacking IL-1, IL-1R1, or IL-18 or receiving IL-1β-neutralizing antibodies and IL-1Ra, which resulted in reduced plaque formation, or in contrast, receiving recombinant IL-18, which exacerbated plaque development (e.g., [218–223]). In contrast with these IL-1 family members, IL-33 or IL-37 exert atheroprotective effects in Apoe^−/−^ mice [224–226].

Notably, insulin resistance, T2D, NAFLD, atherosclerosis, and cardiovascular diseases, each of which are driven in part by IL-1β, are tightly interconnected. Insulin resistance facilitates vascular damage by promoting endothelial dysfunction and inflammation. In turn, atherosclerosis amplifies systemic inflammation, further impairing glucose metabolism and promoting insulin resistance. Additionally, insulin resistance promotes lipogenesis in the liver, which worsens NAFLD, negatively affecting lipid metabolism. This chronic inflammatory loop is further amplified in metabolic syndrome, a condition characterized by the coexistence of obesity, dyslipidemia, insulin resistance, and hypertension. Moreover, IL-1 is a key mediator of the chronic systemic inflammation associated with aging, commonly referred to as inflammaging, which is driven by cellular senescence and immune system alterations [227]. This age-related chronic low-inflammatory state can further contribute to the onset and worsening of the above-described metabolic and cardiovascular diseases [228–230].

Members of the IL-1 cytokine family are also expressed in the central nervous system (CNS), where they contribute to the regulation of neuronal plasticity, immune surveillance and numerous physiological functions, as well as neuroinflammation [167, 231–233].

IL-1 modulates neuronal activity at low concentrations and regulates physiological processes such as synaptic plasticity, sleep, and adult neurogenesis [2, 234]. However, excessive IL-1 activation is implicated in neuroinflammatory and neurodegenerative conditions. For example, IL-1 has been shown to induce neuronal death by stimulating glial cells to release neurotoxic mediators [234].

In Alzheimer’s disease (AD), a condition characterized by the accumulation of amyloid-β (Aβ) peptides and hyperphosphorylated tau aggregates, IL-1 family cytokines have emerged as key players in driving the associated chronic inflammatory state. Elevated IL-1β levels have been observed both in the cells surrounding Aβ plaques in AD animal models and in the cerebrospinal fluid (CSF) of AD patients [235, 236]. In vitro studies have shown that Aβ can directly stimulate astrocytes to release IL-1β and activate the NLRP3 inflammasome [237, 238]. Consistently, genetic ablation of NLRP3 or caspase-1 confers significant neuroprotection in AD mouse models, reducing spatial memory loss and other AD-associated symptoms [239]. Conversely, IL-1Ra-deficient mice show increased vulnerability to neuronal damage following intracerebral injection of human Aβ peptides [240]. In humans, genetic *IL1A* polymorphisms lead to increased expression of IL-1α and have been associated with AD [241]. Therapeutic targeting of IL-1 signaling further supports the pathological role of IL-1 in AD, with the administration of IL-1 receptor-blocking antibodies significantly reducing neuroinflammation and improving cognitive function in AD models [242]. Dysregulation of IL-18 has also been reported in AD. Increased IL-18 plasma levels and elevated IL-18 production by stimulated peripheral blood mononuclear cells have been associated with greater clinical severity, and IL-18 has been found to colocalize with Aβ plaques in the AD brain [243–245]. Despite this evidence, IL-18 plays a protective role in AD experimental models by suppressing aberrant neuronal transmission [246]. IL-33 appears to act in a neuroprotective manner. In AD animal models, IL-33 expression has been associated with reduced disease pathology, whereas increased IL-33 plasma levels have been observed in patients treated with the neuroprotective compound homotaurine [247, 248].

Parkinson’s disease (PD) is characterized by the selective loss of dopamine-producing neurons in the substantia nigra and by the accumulation of fibrillar α-synuclein (aSyn) within the cytoplasm, where it forms Lewy bodies. α-Syn has been shown to directly activate the NLRP3 inflammasome [249]. Moreover, IL-1 has been directly implicated in the neurodegenerative effect of dopaminergic neurons [250], and the administration of IL-1Ra has been shown to attenuate LPS-induced dopaminergic neurodegeneration in mice [251].

Taken together, these findings underscore the complex yet central role of IL-1 family members in mediating sterile acute and chronic inflammation across a broad spectrum of pathological conditions, highlighting their potential as therapeutic targets for cardiovascular, metabolic, and neurodegenerative disorders.

### Autoimmunity and autoinflammation

IL-1 influences nearly all cells and tissues and plays a central role in the pathogenesis of autoimmune and autoinflammatory diseases [2, 3, 36, 252, 253]. Dysregulated or excessive IL-1 activity can drive both localized and systemic inflammatory responses, contributing to autoimmune and autoinflammatory disorders, as well as allergic conditions. Human genetic mutations that lead to excessive IL-1 release or defective IL-1Ra-mediated regulation are associated with autoinflammatory syndromes, which are often characterized by fever, rheumatoid arthritis, psoriatic-like skin conditions, and atherosclerosis [2, 254]. These include genetic mutations in key inflammasome components. Gain-of-function mutations in NLRP3 lead to constitutive inflammasome activation and excessive IL-1β production and are associated with the development of cryopyrin-associated periodic syndrome (CAPS) [255]. Patients with CAPS commonly present with symptoms such as headache, sensorineural deafness, and papilledema, which can be effectively managed with IL-1-targeting therapies. A recent study proposed stratifying patients with CAPS on the basis of their NLRP3 mutations for clinical trials of NLRP3 variant-specific inhibitors [255]. Similarly, gain-of-function mutations in *NLRP1* result in abnormal NLRP1 inflammasome activation and are associated with skin inflammatory diseases and juvenile-onset recurrent respiratory papillomatosis (JRRP) [256–258]. Inherited or somatic mutations in *NLRC4* have also been linked to a spectrum of autoinflammatory disorders, including autoinflammation with infantile enterocolitis (AIFEC) and macrophage activation syndrome (MAS) [259–262]. Furthermore, mutations in the Mediterranean fever gene (*MEFV*), which encodes the pyrin protein, result in increased activation of the pyrin inflammasome and cause familial Mediterranean fever (FMF) [263]. In addition to direct inflammasome components, mutations in upstream regulators can result in similar autoinflammatory conditions. For example, mutations in proline-serine-threonine phosphatase-interacting protein 1 (*PSTPIP1*), which increases its binding to pyrin, promote excessive inflammasome activation and result in pyogenic arthritis, pyoderma gangrenosum, and acne (PAPA) syndrome [264, 265]. Other mutations in genes involved in inflammasome regulation are also associated with immune-mediated pathological conditions. These include loss-of-function variants in dipeptidyl peptidase 9 (*DPP9*), a direct inhibitor of NLRP1 [266–268], and mutations in WD repeat domain 1 (*WDR1*) and cell division cycle 42 (*CDC42*), which alter cytoskeletal dynamics and are linked to aberrant pyrin inflammasome activation [269, 270].

The role of IL-1β in rheumatic diseases is well established, primarily because of the infiltration of myeloid cells into inflamed joints, where they produce IL-1β [254]. IL-1-blocking agents, including canakinumab, anakinra, and rilonacept, have been developed and approved for clinical use in the treatment of autoinflammatory and autoimmune diseases [1, 252–254, 271]. Canakinumab has been approved for conditions such as CAPS, familial Mediterranean fever (FMF), TNF receptor-associated periodic syndrome (TRAPS), and hyper-IgD syndrome (HIDS), also known as mevalonate kinase deficiency (MKD), adult-onset Still’s disease (AOSD), systemic juvenile idiopathic arthritis (SJIA), and gout, whereas anakinra is used to treat CAPS, rheumatoid arthritis, deficiency of interleukin-1 receptor antagonist (DIRA), and COVID-19-related pneumonia. Rilonacept is approved for CAPS, DIRA, and recurrent pericarditis.

Recently, the first clinical case of an autoinflammatory syndrome caused by an autosomal dominant mutation in *IL1R1* was identified in a pediatric patient with chronic recurrent multifocal osteomyelitis (CRMO) [272, 273]. This mutation disrupts the interaction between IL-1Ra and IL-1R1, resulting in a loss of IL-1R1 sensitivity to IL-1Ra (LIRSA) while preserving IL-1α and IL-1β binding. Consequently, unleashed IL-1 signaling triggers a strong inflammatory response characterized by increased production of cytokines such as IL-1β, IL-6, TNF, CXCL8, CXCL1, and CXCL3 by peripheral myeloid cells [272, 273]. On the basis of these findings, a novel IL-1 inhibitor consisting of a fusion protein that selectively traps IL-1β and IL-1α but not IL-1Ra was developed. This inhibitor, named rilabnacept, has been shown to potently suppress IL-1-driven inflammation in vitro and in vivo, including in a model of arthritis [272, 273].

In addition to IL-1, other members of the IL-1 cytokine family, including IL-18, IL-36, IL-33, and IL-37, have been implicated in autoinflammatory and autoimmune diseases. IL-18, which is activated by caspase-1-mediated cleavage, plays a critical role in inflammasome-driven autoinflammatory diseases [274]. In addition to its prominent role as a promoter of type 1 immune responses, in the presence of IL-23, IL-18 promotes IL-17 production by CD4^+^ T cells and γδT cells, contributing to Th17-driven autoimmune pathology [275]. Additionally, IL-18 synergizes with IL-3 to stimulate mast cells and basophils, leading to type 2 cytokine secretion and histamine release. IL-18 has also been shown to regulate skin-resident ILC2s under both steady-state and inflammatory conditions [276]. IL-18 inhibition has demonstrated therapeutic benefits in preclinical models of various inflammatory diseases, and its role has been implicated in several human conditions, including myocardial pathology, metabolic syndrome, inflammatory bowel disease, and macrophage activation syndrome [13, 162].

As discussed above, the cytokine IL-36 is involved in psoriasis and other neutrophilic and inflammatory skin disorders, inflammatory bowel disease, rheumatoid arthritis, and lung inflammation [37]. Genetic polymorphisms in the *IL36Ra* gene or IL-36Ra deficiency are associated with severe psoriatic dermatitis [38].

The IL-33/ST2 axis plays a major role in allergic reactions, particularly in asthma. IL-33 expression is elevated in the bronchial mucosa of asthmatic patients, with levels correlated with disease severity [2]. According to genome-wide association studies, the genes encoding these genes (*IL33* and *IL1RL1*) are associated with asthma susceptibility [277]. Relevant polymorphisms, such as those located in the *IL33* promoter or enhancer, are associated with increased IL-33 production and eosinophilia [278]. In addition to being classical players in type 2 responses, e.g., Th2 lymphocytes, ILC2s, eosinophils, and mast cells, type 2 CD8^+^ cytotoxic cells produce type 2 cytokines in response to IL-33 in experimental allergic airway inflammation, as well as in severe asthma and asthma exacerbations in patients [279]. In addition, genetic analyses have linked IL-33-driven type 2 immunity to tumor progression [280].

A potential link between IL-33 and arthritis has long been suggested, primarily on the basis of correlations between disease severity and IL-33 levels in the synovium, synovial fluid, and circulation [254]. Furthermore, SNPs in *IL33* have been associated with disease severity [281]. Preclinical studies have reported conflicting findings regarding the role of IL-33 in arthritis. Indeed, both the inhibition of IL-33 signaling with an anti-ST2 antibody and the administration of IL-33 have been reported to reduce joint inflammation in mice with collagen-induced arthritis (CIA) [282]. However, IL-33 deficiency did not influence disease severity in mouse models of K/B×N serum transfer-induced arthritis or CIA [283, 284]. These discrepancies suggest that IL-33 may have dual roles in arthritis, acting both as a proinflammatory mediator and as a regulator that promotes anti-inflammatory, Th2-skewed immune responses [254].

## Concluding remarks

IL-1 system ligands and receptors are recognized as key players involved in any condition associated with inflammation and innate or adaptive immune responses, ranging from host responses in infections and sterile tissue damage and inflammation to dysmetabolism, cardiovascular diseases, neurodegeneration and cancer. In addition to the activation of myeloid cells in a proinflammatory mode, IL-1 family ligands are involved in the activation and polarization of innate and adaptive lymphoid cells, highlighting the broadness of the spectrum of activity that mediators of the IL-1 system may play.

Crucially, the system includes several negative regulatory molecules, including antagonists, decoy receptors, and scavengers, that contribute to preventing excessive inflammatory or immune activation mediated by members of the IL-1 system. These regulatory molecules have guided the development of therapeutic tools targeting the IL-1 system. As discussed here, blockade of IL-1 pathways has transformed the management of a spectrum of diseases, ranging from hyperinflammatory conditions associated with infections, such as COVID-19, to chronic immune-mediated disorders and smoldering chronic inflammation that fuels atherogenesis and tumor progression. In addition to anakinra, canakinumab, and rilonacept, new therapeutic anti-IL-1 biologics include gevokizumab, a neutralizing humanized mAb specific to IL-1β, and bermekimab (also known as MABp1), a fully human mAb that targets and neutralizes IL-1a. Furthermore, targeting NLRP3, which affects several IL-1-mediated chronic diseases, including cancer and cardiometabolic and neurodegenerative diseases, is a promising approach since orally available inhibitors have been developed. Under inflammatory conditions, blocking IL-1 with anti-IL-1 biologics is generally associated with a mild increase in infection frequency, particularly for viral respiratory infections, and rarely with neutropenia or hypersensitivity reactions. The intrinsic limitations of these anti-IL-1 drugs include the half-life (very short for anakinra but too long for other anti-IL-1 biologics), the specificity of a single agonist for canakinumab and bermekimab, and the lack of specificity of rilonacept, which scavenges both the agonist and IL-1Ra. NLRP3 inhibitors have a greater safety profile because of their increased specificity and shorter half-life, which limits immune suppression [285]. Furthermore, the new molecular trap Rilabnacept, which selectively recognizes the two agonists and spares IL-1Ra [272, 273], may represent a promising selective approach. The availability of multiple therapeutic approaches to block IL-1 would allow counteracting resistance phenomena observed in selected patients, often due to genetic determinants, including somatic mutations [272, 286]. Continued research into the complexity of the IL-1 system holds promise for refining current therapies and discovering new therapeutic strategies for inflammatory pathologies.
